# Research on Geographical Origin Traceability of *Salvia miltiorrhiza* by Combining Two-Trace Two-Dimensional (2T2D) Correlation Spectroscopy and Improved DeiT Model

**DOI:** 10.3390/plants14213365

**Published:** 2025-11-03

**Authors:** Jinpo Yang, Kai Chen, Yimin Zhou, Jian Zheng, Linhao Sun, Yun Zhang, Zhu Zhou

**Affiliations:** 1 College of Optical, Mechanical and Electrical Engineering, Zhejiang A&F University, Hangzhou 311300, China; yangjp@stu.zafu.edu.cn (J.Y.); ck00@stu.zafu.edu.cn (K.C.); zym20092009@zafu.edu.cn (Y.Z.); 2 College of Food and Health, Zhejiang A&F University, Hangzhou 311300, China; zhengjian@zafu.edu.cn; 3 College of Mathematics and Computer Science, Zhejiang A&F University, Hangzhou 311300, China; sunlh@zafu.edu.cn; 4 Key Laboratory of Agricultural Equipment for Hilly and Mountainous Areas in Southeastern China, Ministry of Agriculture and Rural Affairs, Hangzhou 311300, China

**Keywords:** *Salvia miltiorrhiza* Bunge, 2T2D correlation spectroscopy, data-efficient image transformer, hyperspectral imaging, geographic traceability, convolutional block attention module

## Abstract

*Salvia miltiorrhiza* Bunge (Danshen) is widely used in modern medicine, but the market faces challenges from counterfeit and mislabeled geographical indication products. To address this, we propose a novel framework combining Two-trace Two-dimensional (2T2D) correlation spectroscopy, hyperspectral imaging (HSI), transfer learning, and an enhanced deep learning model (DeiT-CBAM) to identify both authenticity and origin precisely. Hyperspectral data (873–1720 nm) were collected from six genuine and three adulterated regions and converted into synchronous 2T2D correlation spectroscopy images. We systematically evaluated five preprocessing strategies, three wavelength selection methods, three classical models, and four deep learning models. Models based on 2T2D correlation spectroscopy images consistently outperformed traditional one-dimensional spectral models. Notably, the DeiT-CBAM model, integrated with the successive projections algorithm (SPA), achieved optimal performance using only 79 wavelengths, with 100% accuracy on the training and validation sets and 99.62% on the test set, without the need for additional preprocessing. Model interpretability was further validated through layer-wise class activation mapping (layer-wise CAM). This study demonstrates that the integration of synchronous 2T2D correlation spectroscopy images with the DeiT-CBAM model offers robust discriminative performance, providing a reliable technical solution for geographical origin traceability of food, medicinal herbs, and other species.

## 1. Introduction

*Salvia miltiorrhiza* Bunge (Danshen), a perennial herb belonging to the *Lamiaceae* family and the genus *Salvia*, has been widely utilized in traditional Chinese medicine, with its processed roots and rhizomes serving as the principal medicinal parts. In China, it is commonly known as “Danshen”. Traditionally, Danshen has been prescribed for promoting blood circulation, removing blood stasis, relieving pain, calming the mind, alleviating irritability, and cooling the blood to resolve abscesses. Owing to its diverse pharmacological activities, the annual global consumption of Danshen exceeds 20 million km [1]. Codex Alimentarius Commission has positively evaluated the use and safety of Danshen extract, recognizing it as an ingredient in dietary supplements. China represents the major production region, with a cultivation area of more than 40,000 hectares, primarily distributed across provinces such as Shandong, Sichuan, Henan, Shaanxi, and Shanxi [2]. Notably, the concentration of bioactive compounds in Danshen (such as tanshinones and salvianolic acids) exhibits significant regional variation. For instance, research has indicated that Danshen sourced from Shandong province possesses a higher tanshinone content compared to that from Hebei province [3]. Driven by profit, some unscrupulous vendors substitute authentic, geographically indicated Danshen with inferior, non-indicated products. Additionally, counterfeit Danshen—often comprising look-alike herbs such as *Sargentodoxa cuneata* (Oliv.) Rehder & E.H.Wilson, *Arctium lappa* L., and *Dipsacus asperoides* C.Y.Cheng & T.M.Ai—is commonly found in the market [4]. These counterfeit products closely resemble the genuine herb, making visual identification challenging. Consequently, consumer interests are undermined, and market integrity is compromised. Traditional methods for determining the origin of Danshen include inductively coupled plasma mass spectrometry (ICP-MS) [5], isotope ratio mass spectrometry (IRMS) [6], and high-performance liquid chromatography (HPLC) [7]. While these techniques are effective, they are often time-consuming, technically complex, and may damage the samples. Thus, there is a clear need for a rapid, reliable, and non-destructive method to authenticate and trace the origin of Danshen.

Hyperspectral imaging (HSI) integrates spectroscopy with machine vision, enabling simultaneous acquisition of spectral and spatial information, and has been widely applied in food quality control and origin tracing [8,9]. Combined with machine learning, HSI has been used to authenticate camellia seed oil and coarse grain flour [10,11], trace the origin of white peony [12], and identify the sources of turmeric and saffron [13,14]. However, studies focusing on the application of HSI in Danshen are relatively limited. For instance, Jiao et al. used convolutional neural networks (CNN) in conjunction with LIBS to differentiate Danshen from different regions [15], while Dai et al. used HSI with chemometric approaches to predict tanshinone content in Danshen powder and conduct origin classification [16]. Danshen is commonly used in traditional Chinese medicine in the form of slices. Previous geographical origin classification of Danshen required sample pulverization and sieving prior to data acquisition. Therefore, this study investigates whether Danshen slices can be directly measured without pulverization, thus enabling non-destructive detection.

Danshen contains multiple constituents, such as tanshinones, flavonoids, organic acids, and sugars, which often generate complex and overlapping spectral signals, thereby complicating feature extraction [17]. To address this issue, researchers have employed two-dimensional correlation spectroscopy (2DCOS) to analyze spectra under external perturbations, improving spectral resolution and elucidating interrelationships among chemical groups [18]. However, 2DCOS is strongly dependent on the sequence of perturbations. To overcome this limitation, Noda introduced two-trace two-dimensional (2T2D) correlation spectroscopy in 2018, a method capable of constructing two-dimensional spectra using only two one-dimensional (1D) spectra [19]. When combined with chemometrics, 2T2D spectroscopy enables effective discrimination of highly similar samples. The correlated peaks identified in synchronous 2T2D spectra can be used as characteristic variables that influence model performance, thereby facilitating the extraction of critical information and reducing data complexity in spectral modeling.

Deep learning, a rapidly advancing subfield of machine learning, has demonstrated considerable success in diverse analytical tasks, including visual classification and image analysis relevant to medicinal plants, food, and high-value industrial crops [20,21,22,23]. The attention mechanism in deep learning recalibrates feature weighting, enabling models to focus on salient inputs, thereby streamlining data representation and enhancing model efficiency [24]. Within this, the convolutional block attention module (CBAM) effectively captures channel and spatial features while suppressing redundant information, demonstrating good performance in HSI classification. CBAM performs self-attention separately on the channel and spatial dimensions, and then adaptively refines the extracted features [25]. Feng et al. integrated CBAM with Residual Networks (ResNet) to construct a deep convolutional neural network capable of learning features associated with varying disease levels [26]. However, CBAM’s reliance on local characteristics limits its ability to capture global dependencies; consequently, its standalone application may not always yield optimal accuracy. Data-efficient Image Transformer (DeiT) is a deep learning model based on the Transformer architecture [27]. DeiT processes images by segmenting them into patches and projecting these into one-dimensional representations. Its core self-attention mechanism excels at capturing long-range and global feature interactions among these patches. This capability can augment attention mechanisms like CBAM that primarily focus on local features, thereby improving overall feature extraction. Building on the work of Zhang et al. [28], this study proposes that fusing CBAM with DeiT through an attention integration mechanism could enhance the model’s capacity to capture both local and global features, thereby improving classification performance. Furthermore, transfer learning can improve model generalization in small sample scenarios by transferring knowledge—such as feature extraction capabilities—from pretrained models in source domains to target domains, significantly reducing the data requirements for new tasks [29].

**Figure 1:**
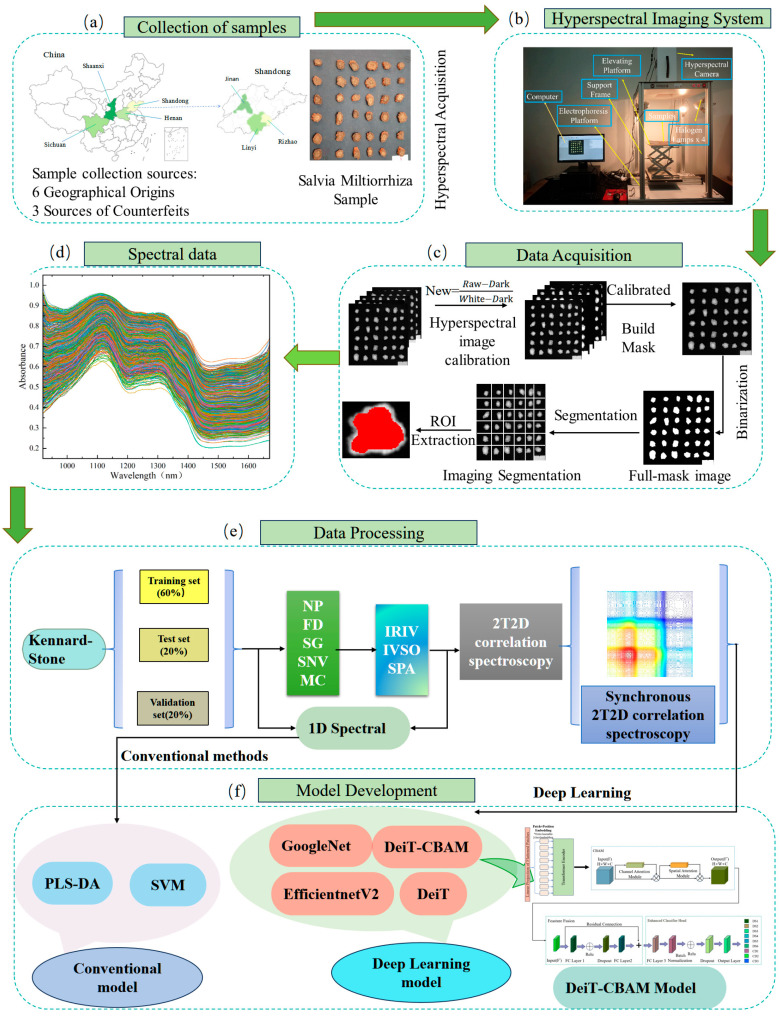
Visualization of the main analytical pipeline for hyperspectral imaging data. Note: (**a**) Sample collection sources; (**b**) Hyperspectral imaging system; (**c**) Spectral data extraction process; (**d**) Spectral data; (**e**) Spectral data processing; (**f**) Model Development; NP: no pre-processing; FD: first derivative; SG: Savitzky–Golay; SNV: standard normal variate; MC: mean centering; SPA: successive projections algorithm; IRIV: iteratively retaining information variables; IVSO: iteratively variable subset optimization.

This study investigates the feasibility of classifying Danshen slices from different geographical origins, as well as their counterfeits, using HSI in combination with transfer learning networks and 2T2D correlation spectroscopy. The research objectives are as follows: (1) to establish the relationship between 2T2D correlation spectroscopy images and Danshen slices from various geographical origins, including counterfeits; (2) to assess the impact of various preprocessing approaches, deep learning models, and wavelength selection strategies on classification performance; (3) to develop a novel classification model—DeiT-CBAM—based on an attention mechanism, aiming to enhance the extraction of both global and local features and improve the model’s ability to learn key 2T2D correlation spectroscopy features of Danshen; (4) to systematically evaluate how the proposed DeiT-CBAM model performs relative to traditional 1D spectral analysis methods and other deep learning models based on 2T2D correlation spectroscopy, with the goal of identifying the optimal classification approach. The overall research framework is illustrated in Figure 1.

## 2. Results

### 2.1. One-Dimensional Spectral Analysis

**Figure 2:**
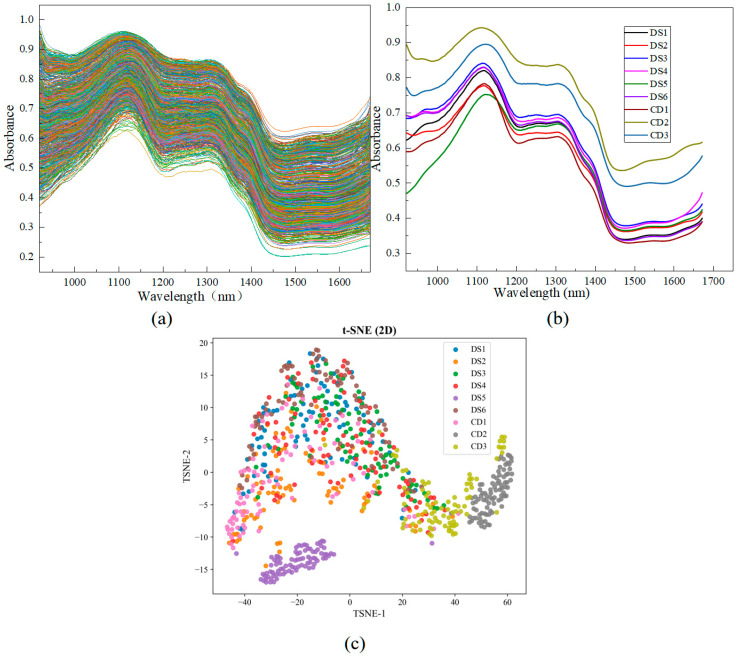
Spectral curves of Danshen and its counterfeits from different origins: (**a**) Spectral curves of the whole samples; (**b**) Average spectral curves; (**c**) The clustering visualization results of the t-SNE analysis.

As illustrated in Figure 2a, the near-infrared hyperspectral curves of all samples exhibited generally consistent patterns. Figure 2b presents the mean spectral curves of Danshen from different geographical origins along with those of its adulterants. Although the sliced samples displayed similar overall spectral trends, notable differences were observed in absorbance. In particular, the mean absorbance values of the adulterants (CD2, CD3) were significantly higher than those of authentic Danshen samples (DS1–DS6). The optimal characteristic wavelength region for tanshinones was identified between 1220 and 1670 nm [30], within which samples DS2 and DS4 exhibited relatively stronger absorbance. The absorption band near 1112 nm may be associated with C–H stretching vibrations or C–C/C=O vibrational modes, primarily related to the aromatic rings and carbonyl structures in phenolic acids and tanshinones; the band at 1308 nm corresponds to the second overtone of C–H stretching vibrations [31], mainly attributed to the methyl/methylene groups on the skeleton of tanshinone compounds; the peak at 1382 nm is likely related to the combination band of C–H bonds in cellulose [32]; and the absorption at 1541 nm is attributed to the first overtone of O–H stretching vibrations, corresponding to characteristic components like phenolic acids in Danshen [33]. Collectively, both authentic and adulterated samples exhibited similar characteristic absorption peaks, resulting in substantial spectral overlap.

To address this overlap and improve the classification accuracy, four spectral preprocessing techniques were applied to the raw spectra. As shown in Figure S1, although preprocessing reduced noise, significant overlapping regions remained, making it difficult to discriminate Danshen samples from different origins. To further elucidate the clustering behavior of high-dimensional spectral data, t-SNE dimensionality reduction was performed on the 453-dimensional dataset (Figure 2c). The results showed that the counterfeit samples (CD1–CD3) formed relatively independent clusters in the two-dimensional space, with CD2 and CD3 clearly separated from authentic Danshen. In contrast, the authentic Danshen samples, with the exception of DS5, exhibited partial overlap due to their geographical proximity and similar spectral characteristics. Therefore, 2T2D spectroscopy was subsequently employed to analyze signal variations in the near-infrared spectra of Danshen. This approach enables the deconvolution of overlapping peaks in complex systems, thereby revealing the relationships among functional groups and elucidating their sequential variations.

### 2.2. Two-Trace Two-Dimensional Correlation Spectroscopy Analysis

**Figure 3:**
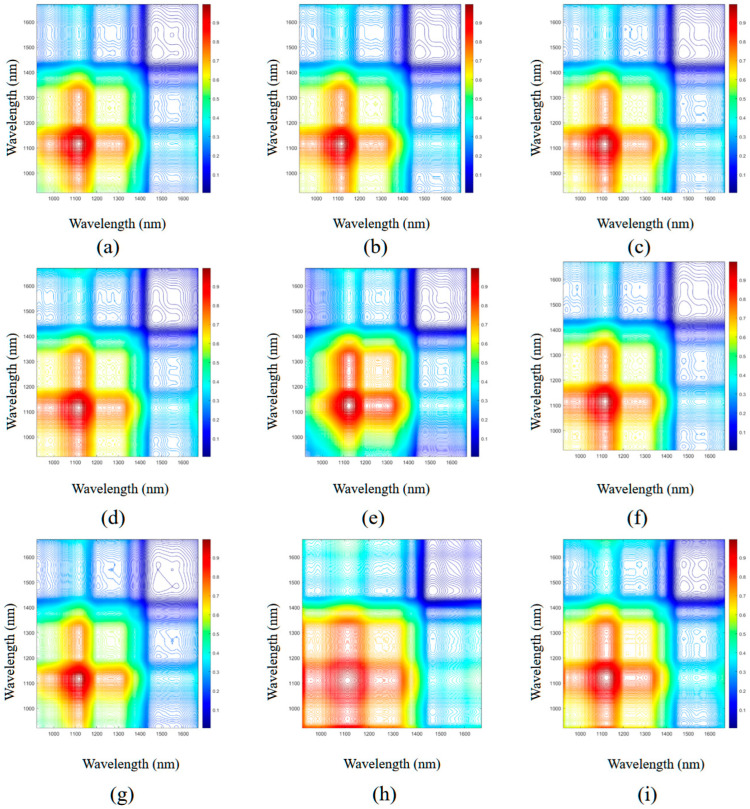
Original full-wavelength synchronous 2T2D correlation spectroscopy of Danshen and its counterfeits: (**a**) DS1, (**b**) DS2, (**c**) DS3, (**d**) DS4, (**e**) DS5, (**f**) DS6, (**g**) CD1, (**h**) CD2, (**i**) CD3.

The synchronous 2T2D correlation spectroscopy of Danshen and its counterfeit samples are presented in Figure 3. These spectra are symmetrical along the diagonal, where the diagonal peaks—known as auto-peaks—result from the autocorrelation of dynamic spectral fluctuations induced by external perturbations [34]. Concentric circles denote auto-peak intensity, with more circles indicating stronger peaks and fewer circles weaker responses. Cross-peaks are off-diagonal structures with positive or negative values. Positive cross-peaks show that spectral intensity change trends at two characteristic wavenumbers are the same under external perturbations, while negative cross-peaks suggest the opposite [35]. Figure 3a–i presents six prominent auto-peaks at 1112, 1203, 1308, 1382, 1458, and 1541 nm, which indicate that these wavelengths are responsive to external stimuli. Among them, 1112, 1308, 1382, and 1541 nm correspond to the main absorption peaks observed in the raw spectra, while 1203 and 1458 nm appear in spectral trough regions. The presence of cross-peaks at combinations such as 1112–1260, 1112–1203, 1112–1308, 1382–1458, and 1038–1541 nm further confirms the synchronous, in-phase behavior of these characteristic wavelengths under perturbation. Variations in the number, location, and intensity of auto-peaks across different samples provide a set of effective spectral features for distinguishing Danshen from its counterfeits and for determining geographical origin. Compared to 1D spectroscopy, 2T2D correlation spectroscopy expands the spectral representation into two dimensions, revealing features that are otherwise difficult to discern and improving spectral resolution. Additionally, it provides inter-wavelength correlation information, offering deeper insight into the structural characteristics of functional components [36].

### 2.3. Analysis of the DeiT-CBAM Model Using Synchronous 2T2D Correlation Spectroscopy

**Figure 4:**
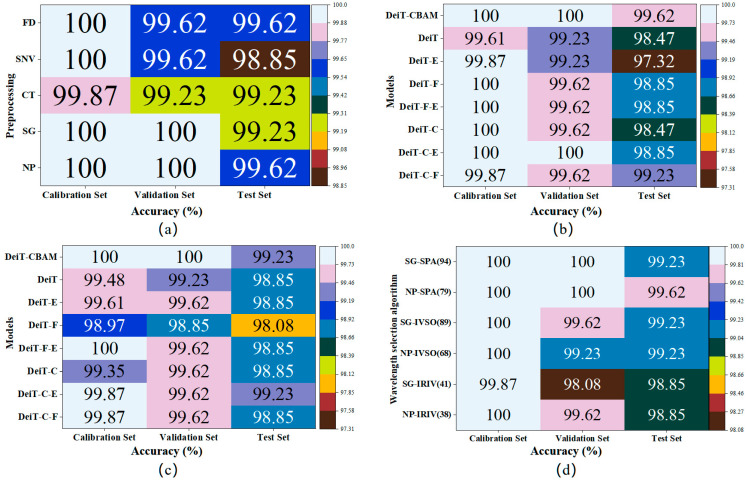
Model performance heatmap: (**a**) Performance comparison of DeiT-CBAM with different preprocessing methods; (**b**) DeiT-CBAM model’s NP spectral ablation results; (**c**) DeiT-CBAM model’s SG spectral ablation results; (**d**) Wavelength selection method performance evaluation. Note: C: CBAM module, F: Feature Fusion, E: Enhanced Classifier Head, NP-IRIV(38): spectrum with no preprocessing method, which, after IRIV wavelength selection, retained 38 wavelengths; other variants follow the same principle.

This study proposed a Danshen and counterfeit classification and identification model based on DeiT-CBAM to improve classification accuracy. Figure 4a presents the classification accuracy of the DeiT-CBAM model with different preprocessing approaches.

The results indicate that the DeiT-CBAM model, by incorporating the CBAM module, enhanced the attention mechanism for channel and spatial features. This enhancement, while retaining the Transformer’s global information processing capability, strengthened the model’s feature extraction capability for localized spectral variations, thus exhibiting good performance in the synchronous 2T2D correlation spectroscopy classification of Danshen and its counterfeits. Experimental data reveal that the DeiT-CBAM model achieved high classification accuracy across all preprocessing methods. In particular, the NP-DeiT-CBAM model reached 100% accuracy on the training and validation sets, with 99.62% accuracy on the external test set.

Analyzing from the perspective of spectral preprocessing, the DeiT-CBAM model using spectra preprocessed by NP and SG reached 100% classification accuracy on both the training and validation sets. In contrast, the classification performance of the DeiT-CBAM model with spectra preprocessed by SNV, FD, and MC exhibited a declining trend. This performance difference may be attributed to these three preprocessing methods, which while enhancing spectral signal quality, unavoidably resulted in significant spectral overlap between classes, thereby reducing the model’s ability to differentiate different classes of Danshen and its counterfeits. The results show that preprocessing methods have an impact on model performance, and this impact can be negative. Considering the influence of preprocessing on model accuracy, subsequent analysis in this study was limited to NP and SG preprocessed spectra.

### 2.4. Ablation Experiment Results

To validate the impact of each module in the DeiT-CBAM model on overall performance, a modular disassembly of the complete model was performed, evaluating the changes in model classification accuracy for synchronous 2T2D correlation spectroscopy of Danshen and its adulterants under different configurations by progressively removing components. The feature fusion module, the CBAM module, and the improved classification head were sequentially removed from the model to investigate the impact of each module on model performance.

Figure 4b,c present the classification accuracy on the training, validation, and external test sets with NP and SG preprocessed spectra after removing each component, along with a comparative analysis of the performance with the complete model. The results indicate that removing any module results in a decline in model performance. The CBAM module demonstrated high test set accuracy in most cases; in the absence of this module, the model accuracy significantly decreased, indicating its substantial impact on model performance. The absence of the Feature Fusion module causes the model to lose its ability to effectively integrate complex spectral features and diminishes its discriminative power for similar spectral patterns, thereby affecting classification performance and model generalization. Although the classification accuracy on the test set decreased to 97.32% when the Enhanced Classifier Head was combined with the DeiT model using original spectra, when integrated with other modules, the Enhanced Classifier Head still made a positive contribution to model performance by consolidating complex features output from other modules through its ReLU activation function and Dropout layer, thereby enhancing the model’s nonlinear expression capabilities. For example, the DeiT-C-E model shows improvements over the DeiT-C model on both the test and validation sets. In conclusion, all modules in the improved DeiT-CBAM model contribute positively to model performance, and the absence of any module leads to a decline in performance.

### 2.5. Characteristic Wavelength Selection

**Figure 5:**
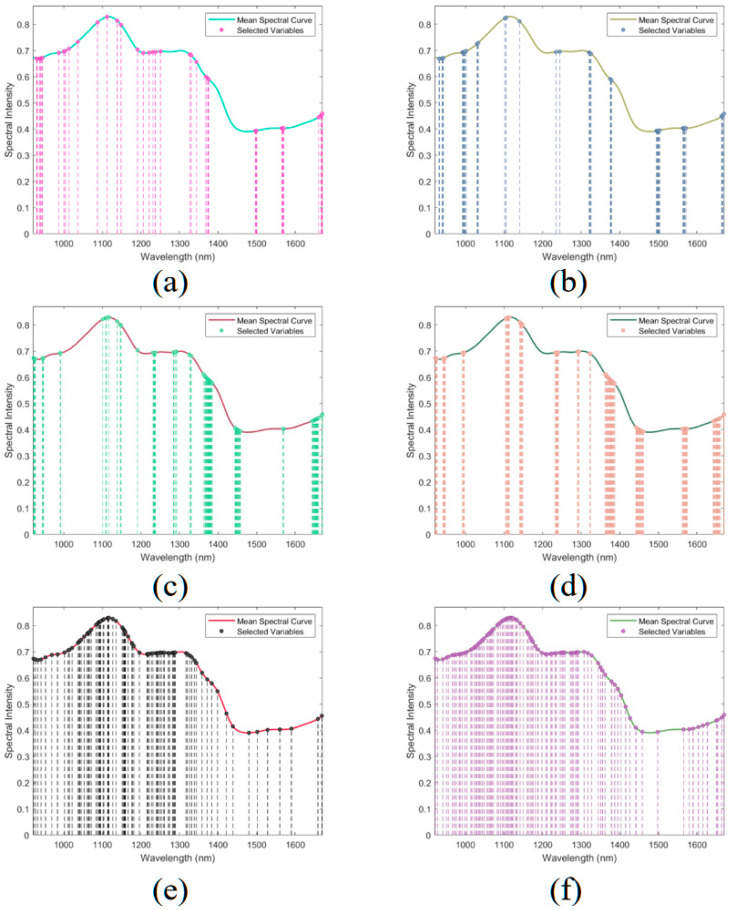
Wavelength distribution after feature selection with various preprocessing methods: (**a**) IRIV + NP, (**b**) IRIV + SG, (**c**) IVSO + NP, (**d**) IVSO + SG, (**e**) SPA + NP, (**f**) SPA + SG.

To enhance the computational efficiency of the DeiT-CBAM model and mitigate the influence of redundant spectral information, this study employed three feature selection algorithms (IRIV, IVSO, and SPA) to extract informative wavelengths from spectra preprocessed using NP and SG methods. The results are illustrated in Figure 5. Owing to disparities in their algorithmic principles, these methods produced notable variations in the chosen wavelengths. Among them, IRIV selected the fewest wavelengths—38 for NP-preprocessed spectra and 41 for SG-preprocessed spectra. This approach predominantly focuses on spectral peaks and troughs, with the chosen wavelengths rather uniformly dispersed throughout the spectrum range. IVSO selected 68 and 89 wavelengths for NP and SG spectra, respectively, concentrating on peak and valley regions while avoiding redundant variables, thus effectively reducing multicollinearity. SPA selected 79 wavelengths for NP and 94 for SG spectra, with some variables located at the spectral boundaries and others corresponding to major peak and valley positions in the spectra.

Figures S2–S4 depict the 2T2D correlation spectroscopy images of NP spectra after wavelength selection using IRIV, IVSO, and SPA. IRIV and IVSO notably reduced cross-peaks, indicating stronger emphasis on extracting independent spectral features and lowering collinearity. In contrast, SPA retained more cross-peaks, suggesting it preserved broader spectral information during dimensionality reduction. The SG results closely resembled those of NP; therefore, only NP-based results are shown.

### 2.6. Analysis of DeiT-CBAM Model Classification Results Based on Feature Wavelengths

Figure 4d presents the classification results of the DeiT-CBAM model employing synchronous 2T2D correlation spectroscopy with three variable selection methods (SPA, IRIV, and IVSO) combined with NP and SG spectral preprocessing techniques. The data indicate that model performance varies significantly depending on the differences in preprocessing methods and feature selection techniques. The NP-SPA-DeiT-CBAM model showed the best performance, relying on only 79 wavelengths, and achieved 100% accuracy on both the training and validation sets, with an accuracy of 99.62% on the external test set. In contrast, the performance of models constructed using IRIV and IVSO methods was relatively weaker; particularly, the SG-IRIV-DeiT-CBAM model only attained an accuracy of 98.08% on the validation set. This may be because the IRIV and IVSO methods eliminated bands containing useful information, resulting in a reduction of model accuracy. Notably, the SPA method, through successive projections and collinearity minimization, more effectively reduces data dimensionality while maintaining stable performance and reducing redundancy.

### 2.7. Comparison of Different Models on the Test Set

To comprehensively evaluate the performance differences between the proposed Synchronous 2T2D correlation spectroscopy combined with DeiT-CBAM model and other approaches—including traditional 1D spectral analysis methods and Synchronous 2T2D correlation spectroscopy integrated with existing deep learning models—their accuracy, precision, recall, and F1 score on the test set were compared. Table S1 presents the classification results of models constructed with traditional 1D spectral methods and deep learning methods that employ 2T2D correlation spectroscopy.

The results presented in Table S1 demonstrate that 2T2D correlation spectroscopy-based models substantially outperform those using 1D spectral data, for both full-band and feature-selected datasets. The PLS-DA model exhibits classification accuracies ranging from 84.11% to 92.34%, whereas the 1D-CNN model achieves accuracies between 89.66% and 93.49%. The SVM model attains accuracies from 87.08% to 94.25%. Comparative analyses reveal that, for geographical traceability and authenticity verification of Danshen samples, both SVM and CNN models surpass PLS-DA in performance. This disparity can be attributed to the pronounced feature similarities among Danshen samples from diverse origins and their adulterants, leading to extensive overlap in sample clusters that exceeds the discriminatory limits of PLS-DA’s linear decision boundaries. Moreover, relative to CNN, SVM exhibits enhanced proficiency in managing highly overlapping features and sustains robust classification efficacy under data-constrained conditions.

All models developed using 2T2D correlation spectroscopy achieved evaluation metrics exceeding 93%. Among them, NP-SPA-DeiT-CBAM (79 wavelengths) and NP-DeiT-CBAM (453 wavelengths) exhibited optimal performance, each reaching accuracy, precision, recall, and F1 score values of 99.62%, 99.63%, 99.62%, and 99.62%, respectively. The NP-IRIV-DeiT-CBAM model (38 wavelengths) exhibited slightly lower performance, with an accuracy of 99.23%. In contrast, traditional CNNs such as GoogleNet and EfficientNetV2 underperformed, with their highest accuracy reaching only 98.85%, likely due to limitations in capturing global dependencies from 2T2D correlation spectroscopy images. Although the DeiT model improved performance via Transformer-based self-attention, its enhancement was modest; only the NP-IVSO-DeiT model achieved 99.23% accuracy, possibly due to insufficient local feature extraction, impacting the discrimination of similar samples.

**Figure 6:**
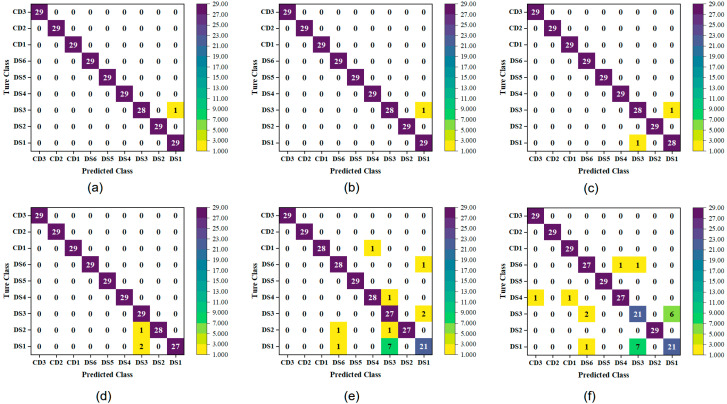
Confusion matrices of different models on the test set: (**a**) NP-DeiT-CBAM, (**b**) NP-SPA-DeiT-CBAM, (**c**) NP-IRIV-DeiT, (**d**) NP-IRIV-EfficientNetV2, (**e**) SG-SVM, (**f**) SG-PLS-DA.

As depicted in the confusion matrix (Figure 6), both the NP-DeiT-CBAM and NP-SPA-DeiT-CBAM models misclassified only a single DS3 sample as DS1. This may be due to the high spectral similarity between samples resulting from their geographical proximity. In addition, the DeiT, GoogleNet, and EfficientNetV2 models exhibited other misclassification issues. For example, the NP-IRIV-EfficientNetV2 model, which performed the best among the EfficientNetV2 models, not only misclassified DS3 samples as DS1, but also misclassified DS2 as DS3. This suggests that the model has weak discriminative ability between certain classes. In contrast, the two traditional models, SG-SVM and SG-PLS-DA, which performed best in 1D spectra, exhibited more significant misclassification issues. Aside from the DS5 samples and the three pseudo-samples (CD1, CD2, CD3), all other categories showed varying degrees of misclassification, especially the misclassification of seven DS1 samples as DS3. This highlights the substantial limitations of traditional methods when dealing with samples that have geographically close origins and minimal spectral differences. Overall, the improved DeiT-CBAM model, combined with 2T2D correlation spectroscopy, can more effectively enhance the discrimination between spectrally similar classes, thus improving overall classification performance.

### 2.8. Feature Activation Map Visualization with DeiT-CBAM

**Figure 7:**
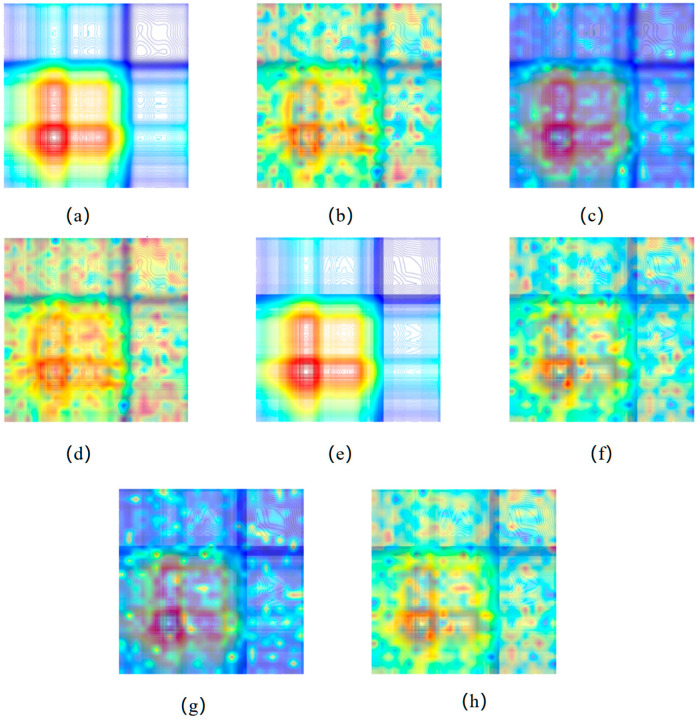
Layer-Wise CAM feature activation map: (**a**) Original 2T2D correlation spectroscopy image; (**b**) DeiT Backbone Feature Activation Map; (**c**) CBAM Module Feature Activation Map; (**d**) Feature Fusion Module Activation Map. (**e**) NP-SPA 2T2D correlation spectroscopy image; (**f**) NP-SPA DeiT Backbone Feature Activation Map; (**g**) NP-SPA CBAM Module Feature Activation Map; (**h**) NP-SPA Feature Fusion Module Activation Map.

To comprehensively assess the effectiveness of the proposed improved DeiT-CBAM model, we employed Layer-wise CAM to visualize feature maps at various stages of the model. As illustrated in Figure 7, we compared the attention heatmaps generated by the model when processing the original full-band 2T2D correlation spectroscopy image and the 2T2D correlation spectroscopy image after SPA feature selection. This comparison includes the outputs from the original DeiT backbone, CBAM, and the feature fusion module. The results demonstrate that, although the original DeiT backbone captures global contextual information through its self-attention mechanism, its activation regions are relatively dispersed. Significant background activation, caused by redundant wavelengths, persists outside the effective signal region (e.g., the top-right quadrant in the figure). This indicates that the model expends additional attention to suppress irrelevant information. Notably, after SPA processing (as shown on the right side of Figure 7f), background activation in the backbone’s heatmap is substantially reduced, and energy is more focused on the core signal region. Upon incorporating the CBAM module, the model’s attention distribution is significantly improved. The red activation region in the bottom-left corner is effectively sharpened and concentrated, especially in the SPA-processed 2T2D correlation spectroscopy image, where the focusing effect of CBAM is more precise. This indicates that CBAM optimizes the model’s attention, focusing on the core areas of local features, thus enhancing attention to discriminative local features. The output feature map after the feature fusion module showcases the synergistic effect of both global and local attention. It preserves the highly concentrated core activation region introduced by CBAM (the red region in the bottom-left corner) while re-establishing the DeiT backbone’s perception of global context. By effectively integrating global and local attention information across different scales, the final feature representation becomes more comprehensive and balanced. The consolidated results from these visualizations indicate that our model improves feature extraction quality by combining the global modeling advantage of Transformers with the local perceptive capabilities of CBAM. Furthermore, the integration of the SPA-processed 2T2D correlation spectroscopy image strengthens this advantage from the data source, offering an inherent explanation for the observed enhancement in the model’s classification performance.

## 3. Discussion

The findings indicate that the effective selection of wavelength variables reduces redundancy in the original dataset. However, neglecting particular spectral details may impair the model’s efficacy, highlighting the importance of employing appropriate variable selection techniques and modelling methodologies to develop high-accuracy models [37]. The NP-SPA-DeiT-CBAM model maintains the accuracy and precision of predictions while reducing the number of wavelengths from 453 to 79, significantly simplifying the model structure. Through the optimization of wavelength selection via SPA, the DeiT-CBAM model is able to significantly reduce computational complexity while retaining high predictive performance. Converting the 1D spectra into 2T2D correlation spectroscopy effectively addresses the issue of spectral overlap and reveals differences between Danshen from different geographic origins and their counterfeit counterparts.

The 2T2D correlation spectral method effectively converts subtle chemical differences driven by geographical origin into clear, visually interpretable features that can be efficiently learned by deep learning models. Geographical origin encompasses a range of complex environmental variables, such as soil mineral content, climate, and altitude, all of which directly influence the plant’s secondary metabolism. This leads to small but consistent changes in the relative content of active components (such as tanshinones and salvianolic acids) and structural components (such as cellulose). In traditional one-dimensional near-infrared (NIR) spectroscopy, these differences are often masked. The NIR region inherently belongs to a broad range of frequencies and harmonic bands (such as C–H, N–H, O–H), where multiple chemical components cause significant spectral overlap, making it challenging to separate them effectively. The 2T2D correlation spectral method transforms the one-dimensional spectrum into a two-dimensional correlation image, shifting the analysis from simple absorption intensity to the synchronous variation between different spectral bands [38]. For example, slight changes in the ratio of phenolic acids to cellulose caused by geographical factors may not be significant in a one-dimensional spectrum. However, in the 2T2D correlation image, this is manifested as a noticeable change in the cross-peak intensity or shape of the characteristic bands associated with these components (such as the peaks at 1541 nm and 1382 nm). These cross-peaks effectively amplify subtle chemical changes.

Compared with EfficientNetV2, GoogLeNet, and the original DeiT model, the improved DeiT-CBAM model effectively integrates the CBAM attention mechanism into the Transformer architecture. By incorporating an optimized feature fusion structure and an enhanced classification head, it achieves a balanced extraction of global and local features. This design enables more precise capture of spatial and spectral information, thereby identifying key discriminative features in the 2T2D correlation spectroscopy of Danshen and substantially enhancing the model’s ability to differentiate samples from geographically adjacent origins. Consequently, the model is better suited for classifying the geographical origin of Danshen. The integration of 2T2D correlation spectroscopy with HSI converts 1D spectral data into information-rich two-dimensional synchronous correlation spectral images, thereby improving the model’s feature extraction capabilities. Furthermore, transfer learning facilitates knowledge transfer from source to target tasks, mitigating challenges associated with limited computational resources and data scarcity. This approach reduces model overfitting while preserving high training efficiency under data constraints. The transfer learning framework, based on 2T2D correlation spectroscopy combined with deep learning, exhibits considerable potential for accurate classification and reduced computational complexity. Comparisons with traditional methods and other deep learning models further confirm the robustness and efficiency of the proposed approach, underscoring its applicability in tracing the origin of Danshen, identifying other traditional Chinese medicinal materials, and its potential in food quality control and other related fields.

However, it must be acknowledged that there are still areas in this research that require further improvement. Expanding the dataset by incorporating Danshen samples from various geographical regions will enhance the model’s effectiveness and adaptability. Future efforts will focus on refining data processing methods for varieties with similar geographical origins, enabling the model to capture more intricate features and improve classification performance. Additionally, research will aim to optimize the model’s scalability to better meet the demands of practical applications.

## 4. Materials and Methods

### 4.1. Sample Preparation

In June 2024, we conducted sample collection, covering the main production areas of *Salvia miltiorrhiza* (Danshen) across four provinces in China [39]. Additionally, to enhance the model’s ability to differentiate subtle geographical variations, three representative sub-areas within the primary production region of Shandong Province were selected. In total, samples were collected from six different regions. Three commonly confused medicinal plants—*Dipsacus asperoides*, *Sargentodoxa cuneata*, and *Arctium lappa*—were also included as controls. Table S2 summarizes the sample codes, species names, and collection locations. From each collection area, 48 plants with similar growth conditions were selected for sampling. All samples were transported under refrigerated conditions (4 °C) to the laboratory, where side roots and adventitious roots were removed, and only the main roots were retained. After eliminating excess impurities, a 3 mm-thick slice was taken from the upper, middle, and lower segments of the main root for analysis. These slices were then placed in a drying oven (DHG-9143BS-111, Shanghai Jiehan Testing Equipment Co., Ltd., Shanghai, China) at 55 °C until the weight remained unchanged after two consecutive measurements (with a change of less than 0.1%). In total, nine categories were established (six Danshen production regions and three controls), comprising 1296 samples (144 slices per category). Given the large number of samples and the complex, time-consuming procedure, all samples were stored in a 25 °C drying oven prior to testing to prevent oxidation, moisture absorption, or air contamination.

### 4.2. Hyperspectral Image Acquisition and Calibration

All samples were scanned using a near-infrared hyperspectral imaging (HSI) system (GaiaField-N17E-HR, HSIA-BD; Sichuan Shuangli HePu Technology Co., Ltd., Chengdu, China), which includes an imager, enclosed testing chamber, four halogen lamps (50 W each), a lifting platform, motorized displacement stage, and computer, as shown in Figure 1b. Image acquisition was controlled via SpecVIEW software (3.1.259 version), with the lens positioned 35 cm above the sample and an exposure time of 5.7 ms. Halogen lamps were preheated for at least 30 min to ensure stable illumination. Each sample was scanned against a black background, and 512 spectral bands (873–1720 nm, 1.57 nm resolution) were collected. To correct for image noise and non-uniform illumination, hyperspectral images were calibrated using black-and-white reference images, and the relative reflectance was computed as follows:

(1) $$R=\frac{I-D}{W-D}\times 100\%$$

where *I* is the original image, *W* is the white reference, and *D* is the dark reference. *D* was captured by covering the lens with a cap (reflectance ~0%), while *W* was acquired using a white PTFE (polytetrafluoroethylene) panel with 99% reflectance.

### 4.3. Spectral Extraction

As shown in Figure 1c, region of interest (ROI) extraction from hyperspectral images involved selecting a clear grayscale image at 1046 nm for binarization, followed by masking and morphological operations (dilation, erosion, and thresholding at 0.2) to isolate the ROI. Reflectance spectra were converted to absorbance spectra using the Beer–Lambert law: *A* = *log*(1/*R*). A total of 1296 spectra were obtained and divided into training, validation, and test sets (3:1:1) using the Kennard–Stone (KS) method. Due to noise interference below 920 nm and above 1670 nm, only the 920–1670 nm range was used for analysis, yielding 453 valid spectral bands.

### 4.4. Data Preprocessing and Feature Wavelength Selection

To minimize the influence of both the intrinsic properties of the samples and environmental factors during spectral acquisition, this study employed five preprocessing strategies: no preprocessing (NP), standard normal variate (SNV), Savitzky–Golay (SG), mean centering (MC), and first derivative (FD). MC was applied to eliminate feature bias and reduce multicollinearity [40]. SG was used to smooth spectral signals and suppress high-frequency noise [41]. In this study, the filter window size was set to 11, and the polynomial order was set to 3. SNV was employed to correct for scattering effects and mitigate interference from particle size variations. FD was typically used to remove vertical shifts and correct baseline drift caused by linear trends [42,43].

The high dimensionality of spectral data poses computational challenges and hampers processing efficiency [44]. To address this, three widely used feature wavelength selection methods—Successive Projections Algorithm (SPA), Iteratively Retaining Informative Variables (IRIV), and Iterative Variable Subset Optimization (IVSO)—were employed to reduce redundant information, enhance model simplicity, improve computing efficiency, and boost predictive accuracy. SPA selects wavelengths with minimal collinearity via vector projections, identifying variables with maximum variance in the orthogonal subspace [45]. In this study, the number of selected wavelengths was constrained to between 10 and 100. IRIV recursively removes redundant variables while retaining those with high model contribution, combining variable importance ranking and model evaluation to improve efficiency without compromising accuracy [46]. In this study, 5-fold cross-validation and partial least squares (PLS) with a maximum of 10 latent variables were employed to optimize the selection of informative features. IVSO, also PLS-based, integrates weighted sampling and variable ranking by building multiple sub-models, evaluating variable importance, and iteratively adjusting weights based on prediction error. The optimal subset is determined by selection frequency, balancing predictive performance with computational cost [47]. In this study, IVSO parameters included 5-fold cross-validation and 5000 sampling runs using WBMS.

### 4.5. Two-Trace Two-Dimensional Correlation Spectra Acquisition

Two-trace two-dimensional (2T2D) correlation spectroscopy is a method designed to extract detailed information by comparing a pair of spectra. This study utilizes 2T2D to analyze the variations between an individual sample and the average of its group. For this analysis, the individual spectrum was designated as the sample spectrum, s(v), and the group average spectrum was used as the reference spectrum, r(v).

According to Noda’s 2T2D theory, the synchronous spectral intensity, $\Phi (v_{1},v_{2})$, highlights similar patterns of intensity variation between the two spectra. The expression for this is:

(2) $$\Phi (v_{1},v_{2})=\frac{1}{2}[s(v_{1})\cdot s(v_{2})+r(v_{1})\cdot r(v_{2})]$$

In this study, MATLAB 2023b was used to generate synchronous 2T2D correlation spectroscopy images for 1296 samples (Danshen and counterfeits) within the 920–1670 nm range. To reduce noise and ensure uniformity during model training, all images were min–max normalized, resized to 600 × 600 pixels in PNG format, and sorted into folders for analysis.

### 4.6. Model Development and Evaluation

#### 4.6.1. Conventional One-Dimensional Spectral Models

Three classification algorithms—support vector machine (SVM), partial least squares-discriminant analysis (PLS-DA), and one-dimensional convolutional neural network (1D-CNN)—were employed to construct models using both full-band and feature-selected 1D spectral data. SVM maps non-linearly separable data into a higher-dimensional space via kernel functions, where an optimal hyperplane with soft margins enables robust classification [48]. In this study, the radial basis function (RBF) kernel was employed. PLS-DA treats classification as a regression task by applying partial least squares regression to maximize covariance between predictors and class labels, followed by a threshold (0.5) to convert predicted values into class assignments [49,50,51].

The custom-designed 1D-CNN consists of two parts: feature extraction and classification. The feature extraction part is composed of two one-dimensional convolutional layers and their corresponding pooling layers. First, the input 1D spectral features pass through the first convolutional layer with ReLU activation, followed by dimensionality reduction through a max pooling layer. Next, the second convolutional layer further extracts deep features, again using ReLU activation and max pooling. To mitigate the risk of overfitting, a Dropout layer is added after each convolutional and pooling operation. The extracted feature vectors are then flattened and input into a two-layer fully connected network: the first layer maps the features to a 64-dimensional space (with ReLU activation), and the final output layer consists of 9 neurons, performing the 9 class classification task.

This research applied grid search to identify optimal hyperparameter configurations for all models [52].

#### 4.6.2. Transfer Learning Models

The transfer learning process begins with initializing models using pre-trained weights, followed by fine-tuning to adapt to a new classification task. Typically, the final fully connected layer is replaced to match the number of target classes—in this study, 9 neurons representing 9 categories of Danshen and its counterfeit samples. Four pre-trained models—GoogleNet, EfficientNetV2, DeiT, and a modified DeiT-CBAM—were employed. Classification models were built using both full-wavelength and feature-wavelength 2T2D correlation spectroscopy images. Leveraging general features learned from the large-scale ImageNet dataset, these models were fine-tuned to improve task-specific performance, reduce training time and computational costs, and enhance generalization. A brief overview of each model is provided below:

GoogleNet [53], introduces the Inception module to capture multi-scale features efficiently, enabling high classification performance with relatively low computational cost. EfficientNetV2 [54] improves training speed and parameter efficiency through MBConv/Fused-MBConv modules and a progressive learning strategy. DeiT [27], introduces a data-efficient Vision Transformer with distillation token learning, achieving strong classification performance using only ImageNet and enabling efficient global feature modeling through self-attention. Detailed architectural principles of all models are described in the respective original publications.

**Figure 8:**
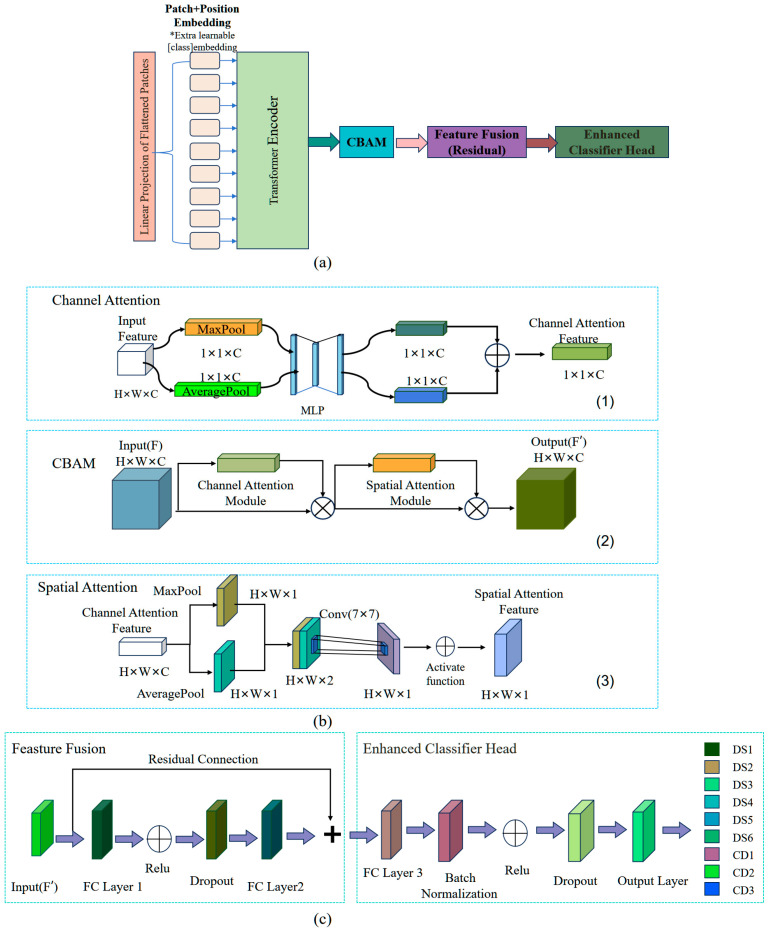
Architecture of the DeiT-CBAM model: (**a**) Overall structure of the DeiT-CBAM model: (**b**) Structure of the CBAM module: (**c**) Feature fusion and enhanced classifier head architecture.

To improve feature extraction and classification accuracy, a transfer learning model combining DeiT with the CBAM module is proposed (Figure 8a). By integrating DeiT’s self-attention with CBAM, the model effectively captures both global and local spectral features essential for geographic origin classification based on 2T2D correlation spectroscopy, thus enabling focus on salient information while retaining key spectral details. A Feature Fusion module with residual connections further enhances representation, generalization, and training stability. An optimized classification head improves expressiveness while reducing overfitting risk.

As illustrated in Figure 8b, CBAM enhances the model’s ability to focus on salient features by incorporating attention mechanisms in both spatial and channel dimensions, while effectively reducing interference from irrelevant information [55]. In the channel attention mechanism of CBAM, the input features are subjected to global max pooling and average pooling independently to extract salient features and contextual information. The aggregated outcomes from both pathways are subsequently input into a shared-weight two-layer multilayer perceptron (MLP). Following element-wise addition for fusion, a weight vector is produced, which is subsequently activated by a Sigmoid function to generate the channel attention map. The computational sequence of the channel attention mechanism is outlined as follows:

(3) $$M_{c}(F)=f_{sigmoid}(MLP(AveragePool(F))+MLP(MaxPool(F)))$$

The spatial attention mechanism enhances the model’s focus on critical image regions by optimizing spatial information in feature maps. The processing pipeline is as follows: First, the output of channel attention mechanism undergoes element-wise multiplication with the original feature map to generate enhanced input representations. Subsequently, max pooling and average pooling operations are separately applied to these representations, yielding two distinct feature maps. These feature maps are then concatenated along the spatial domain and processed through a 7 × 7 convolutional layer to capture spatial dependencies. A Sigmoid activation function is ultimately employed to generate the spatial attention map. This process is computed as follows:

(4) $$M_{s}(F)=f_{\mathrm{sigmoid}}(\mathrm{Conv}([\mathrm{AveragePool}(F);\mathrm{MaxPool}(F)]))$$

The combination of both enhances the model’s expressive capacity across spatial and channel dimensions. The resulting weighted features are then fed into the feature fusion module, where a non-linear transformation is applied via two fully connected layers. To retain original feature information, residual connections are incorporated. The fused features are subsequently passed through a Dropout layer to mitigate overfitting and improve model stability. The classification head adopts a multi-layer architecture comprising a hidden layer with 512 nodes, along with Batch Normalization and Dropout layers, to boost classification accuracy and enhance generalization performance. The overall structure is illustrated in Figure 2c.

To ensure a fair comparison across all pre-trained models, hyperparameter settings were standardized for all experiments. Based on preliminary testing, cross-entropy was selected as the loss function [56], and network weights were optimized using Adaptive Moment Estimation with Decoupled Weight Decay (AdamW) [57], an improved version of Adam that incorporates weight decay to enhance generalization. $\beta_{1}$ was set to 0.9, with an initial learning rate of 0.001 that reduced by a factor of 0.8 every 10 iterations. These hyperparameter settings aimed to accelerate learning in early stages and promote convergence as the learning rate gradually decreased. The transfer learning model was trained on five categories of 2T2D correlation spectroscopy images—original, FD-, SG-, MC-, and SNV-preprocessed—to evaluate and compare their classification performance.

#### 4.6.3. Model Evaluation

The model’s reliability and stability were evaluated using Accuracy, Precision, Recall, and F1 score. The procedure for calculating these metrics is described below:

(5) $$\mathrm{Accuracy}=\frac{TP+TN}{TP+FP+FN+TN}$$

(6) $$\mathrm{Precision}=\frac{TP}{TP+FP}$$

(7) $$\mathrm{Recall}=\frac{TP}{TP+FN}$$

(8) $$F1\;\mathrm{score}=\frac{2\times \mathrm{Precision}\times \mathrm{Recall}}{\mathrm{Precision}+\mathrm{Recall}}$$

The correctly and wrongly categorized positive examples are denoted by TP and FP, respectively, and the correctly and incorrectly classified negative instances by TN and FN. For each evaluation metric, a higher value indicates better model performance.

### 4.7. Visualization Method

To elucidate the improved decision-making mechanism within the DeiT model in this study, we adopted a hierarchical class activation mapping (Layer-wise CAM) method [58]. This method is based on the classic Gradient-weighted Class Activation Mapping (Grad-CAM) approach [59], which has been extended to multiple depth layers within the network. The goal is to reveal the evolution of features at different processing stages of the model. The core principle of this method is as follows: the importance weight $\alpha_{k}^{c}$ for each feature channel is calculated by taking the gradient of the prediction score for a specific class $y^{c}$ with respect to the feature map $A^{k}$ at the target layer. These weights are obtained by performing global average pooling on the gradients. Ultimately, the heatmap $L_{\mathrm{Grad}\text{-}\mathrm{CAM}}^{c}$ is generated by performing a weighted sum of the feature maps $A^{k}$, followed by the application of a ReLU activation function, as shown in Equation (9).

(9) $$L_{\mathrm{Grad}-\mathrm{CAM}}^{c}=\mathrm{ReLU}\left(\sum_{k}\left(\frac{1}{Z}\sum_{i}\sum_{j}\frac{\partial y^{c}}{\partial A_{ij}^{k}}\right)A^{k}\right)$$

In the DeiT backbone network, the Grad-CAM method was adapted to the output sequence of the final Transformer block. The resulting 1D activation vector was reshaped into a 2D heatmap corresponding to the original image patch layout, enabling the identification of key image feature regions.

For the CBAM module and Feature Fusion module, the 1D feature vector output was reshaped into a 2D feature map, followed by application of the standard Grad-CAM process. This enabled visualization of category-specific features both after the CBAM module and prior to classification following the Feature Fusion module.

Meanwhile, to further substantiate the clustering characteristics of different sample categories within the high-dimensional spectral space, this study employed t-distributed stochastic neighbor embedding (t-SNE) for dimensionality reduction and subsequent visualization of the original spectral features [60].

### 4.8. Software Tools

MATLAB R2023b (MathWorks, Natick, MA, USA), ENVI 5.3 (Exelis Visual Information Solutions, Boulder, CO, USA), Python 3.11.0, PyTorch 2.6.0+cu126, torchvision 0.21.0+cu126, and Origin 2022 (OriginLab Corporation, Northampton, MA, USA) were used to analyse the data. Every piece of software was run on a Windows 11 computer with a 3.2 GHz Intel Core i9-14900KF processor, 64 GB of RAM, and two NVIDIA RTX 4090 GPUs with 24 GB of video memory each.

## 5. Conclusions

This study proposes an integrated approach combining Near-Infrared Hyperspectral Imaging (NIR-HSI), deep learning, transfer learning, and the 2T2D correlation spectroscopy algorithm for the rapid, non-destructive authentication and geographic origin identification of Danshen slices. The DeiT-CBAM model, which synergistically integrates the attention mechanisms of CBAM and DeiT, effectively captures both global and local spectral features, showcasing superior performance in Danshen origin classification. Under the framework of synchronous 2T2D correlation spectroscopy, the study systematically evaluated the combined effects of five spectral preprocessing methods, three variable selection techniques, and four deep learning transfer learning models. The results demonstrated that the optimized DeiT-CBAM model, integrated with synchronous 2T2D correlation spectroscopy images, delivered outstanding classification performance. Specifically, without any data preprocessing and using only SPA wavelength selection, both the training and validation sets achieved 100% accuracy, while the test set achieved an accuracy of 99.62%. Furthermore, the integration of the optimized DeiT-CBAM model with synchronous 2T2D correlation spectroscopy and transfer learning offers significant potential for applications in geographic traceability and quality assessment and ensuring the authenticity and reliability of other high-value medicinal plants and economic crops, playing a vital role in product integrity and quality assurance.

## Data Availability

The original contributions presented in the study are included in the article/Supplementary Materials; further inquiries can be directed to the corresponding authors.

## Supplementary Materials

The following supporting information can be downloaded at: https://www.mdpi.com/article/10.3390/plants14213365/s1, Figure S1: Average spectral curves after preprocessing; Figure S2:Combine the 2T2D correlation spectroscopy image with the IRIV feature wavelength selection algorithm; Figure S3: Combine the 2T2D correlation spectroscopy image with the IVSO feature wavelength selection algorithm; Figure S4: Combine the 2T2D correlation spectroscopy image with the SPA feature wavelength selection algorithm; Table S1: Performance of different classification models on the test set; Table S2: Collection sources of Danshen and its adulterants.
