# Supplementary material for: Research on Geographical Origin Traceability of Salvia miltiorrhiza by Combining Two-Trace Two-Dimensional (2T2D) Correlation Spectroscopy and Improved DeiT Model
Source: Plants (Basel). 2025 Nov 3;14(21):3365. doi: 10.3390/plants14213365 (PMC12608159; doi:10.3390/plants14213365)
Supplement: Supplementary file 1 [file plants-14-03365-s001.zip › plants-3916233-supplementary.pdf]

Supplementary material

Fig. S1. Average spectral curves after preprocessing.

Fig. S2. Combine the 2T2D correlation spectroscopy image with the IRIV feature wavelength selection algorithm.

Fig. S3. Combine the 2T2D correlation spectroscopy image with the IVSO feature wavelength selection algorithm.

Fig. S4. Combine the 2T2D correlation spectroscopy image with the SPA feature wavelength selection algorithm.

Table. S1 Performance of Different Classification Models on the Test Set.

Table. S2 Collection Sources of Danshen and Its Adulterants

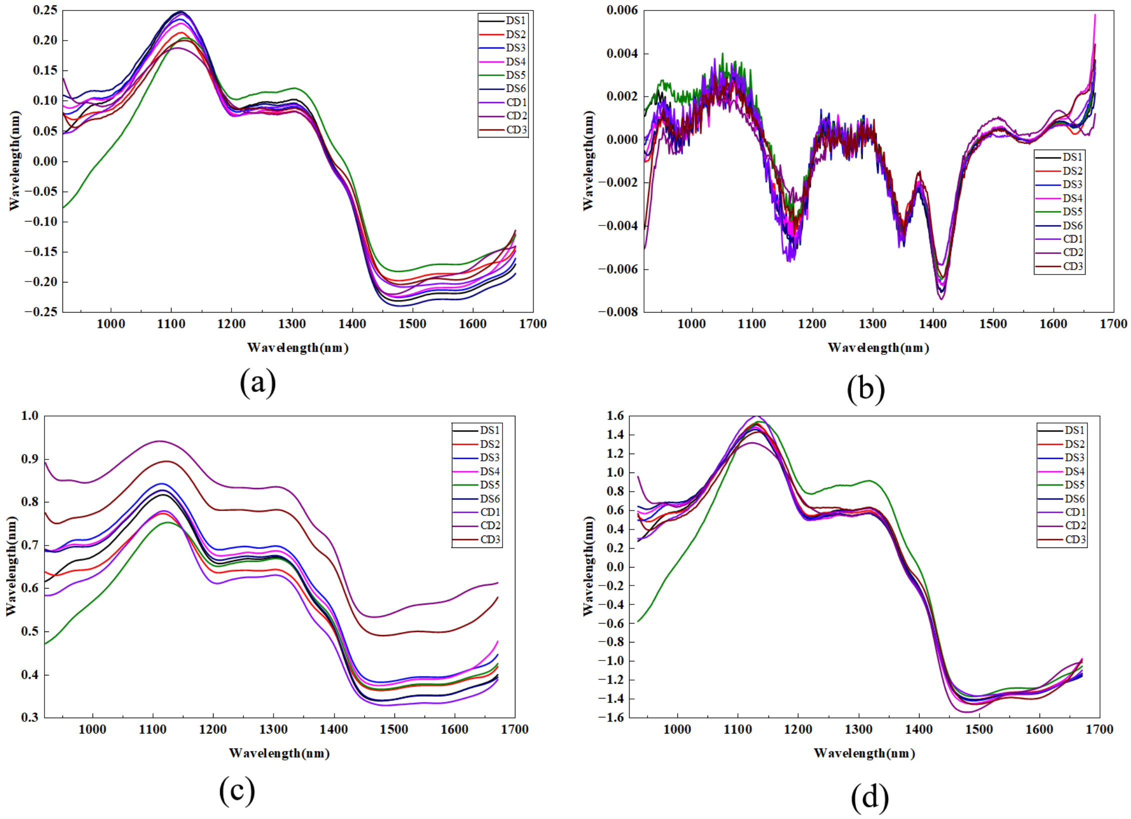

Fig. S1. Average spectral curves after preprocessing: (a) MC, (b) FD, (c) SG, (d) SNV.

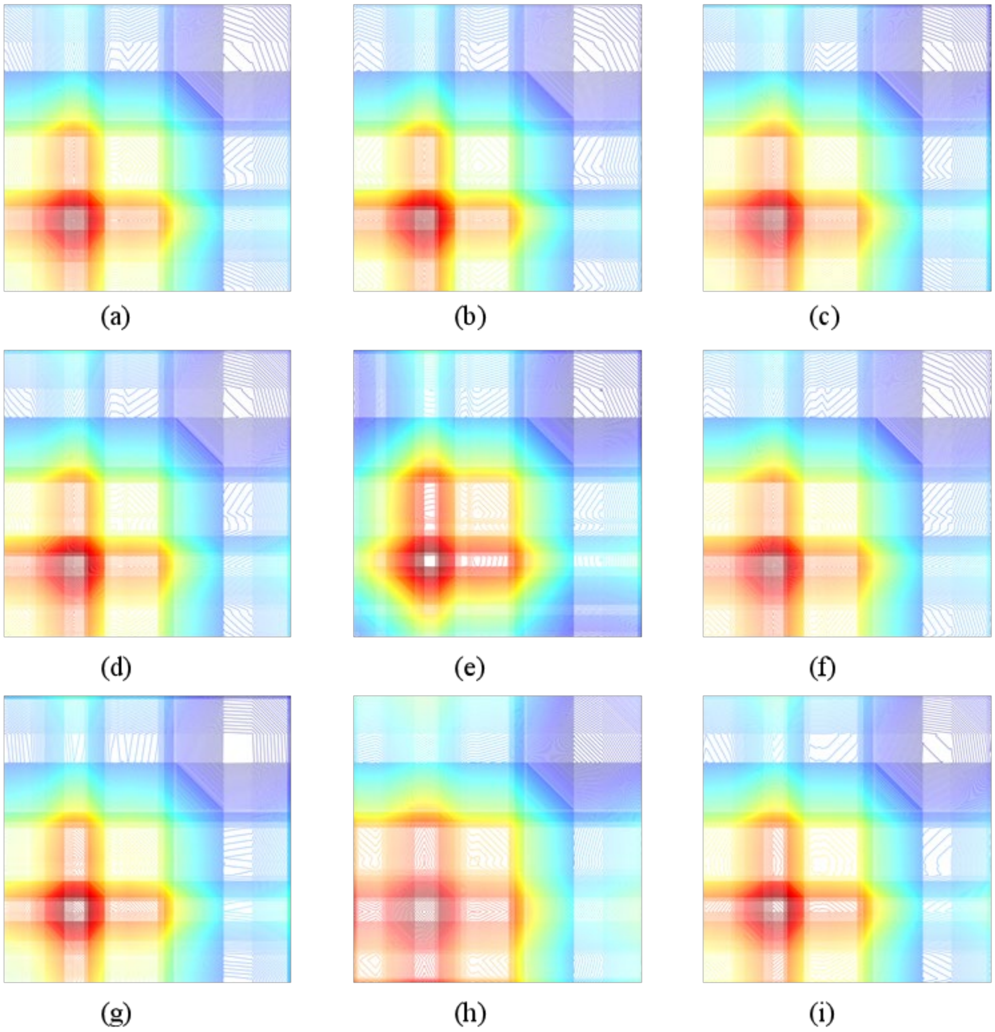

Fig. S2. Combine the 2T2D correlation spectroscopy image with the IRIV feature wavelength selection algorithm: (a) DS1, (b) DS2, (c) DS3, (d) DS4, (e) DS5, (f) DS6, (g) CD1, (h) CD2, (i) CD3.

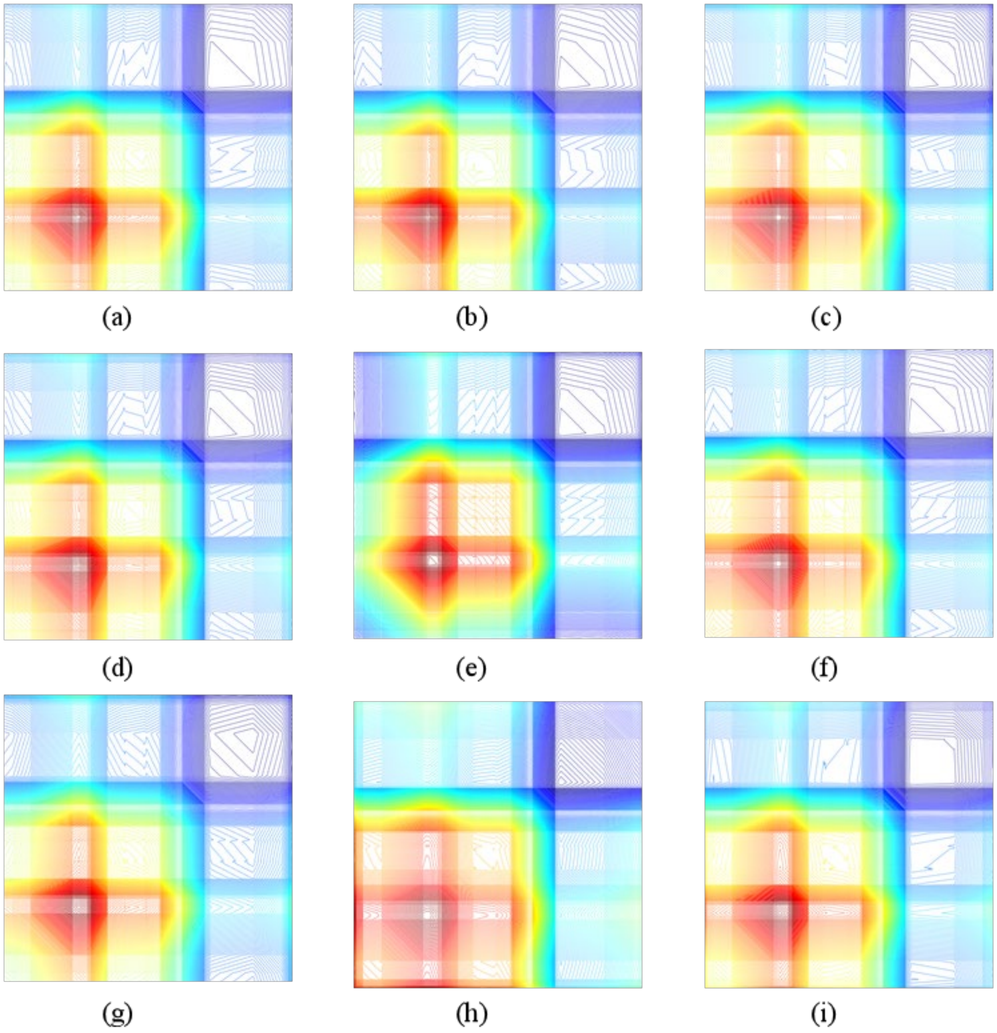

Fig. S3. Combine the 2T2D correlation spectroscopy image with the IVSO feature wavelength selection algorithm: (a) DS1, (b) DS2, (c) DS3, (d) DS4, (e) DS5, (f) DS6, (g) CD1, (h) CD2, (i) CD3.

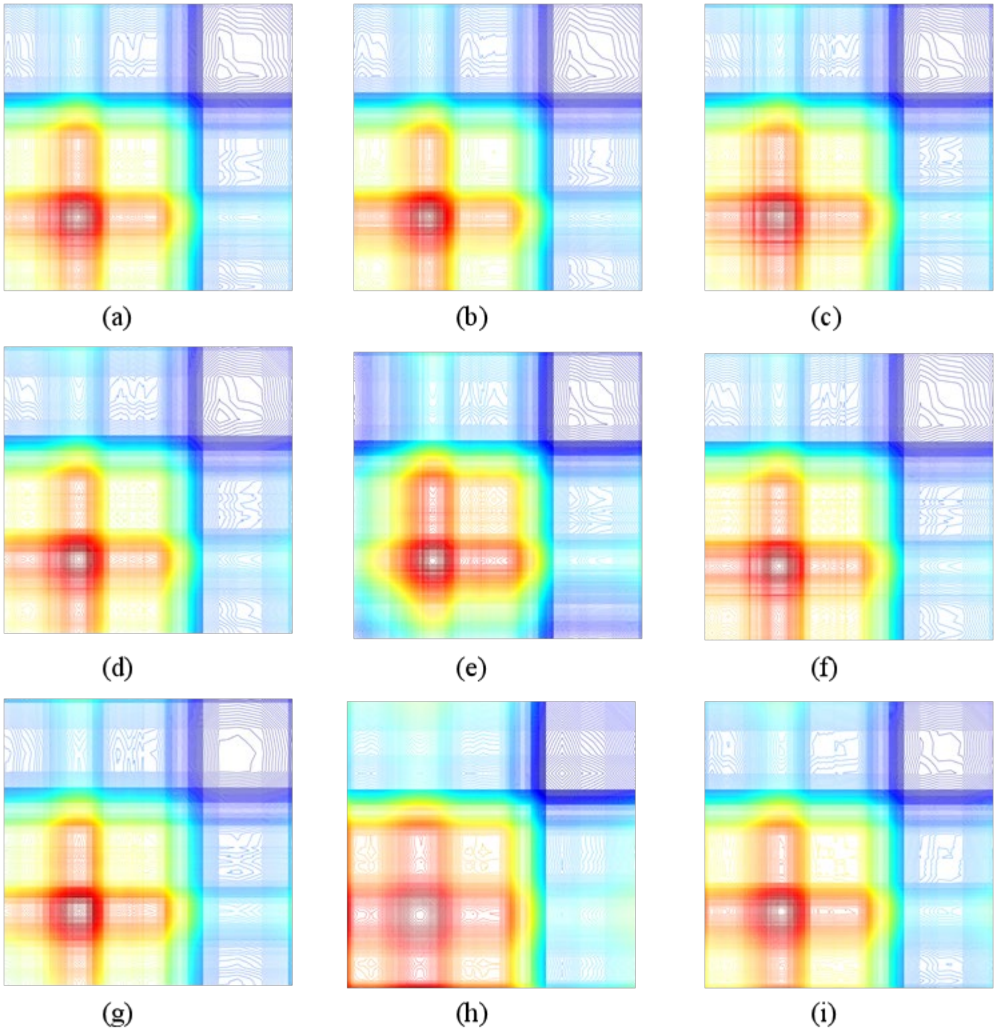

Fig. S4. Combine the 2T2D correlation spectroscopy image with the SPA feature wavelength selection algorithm: (a) DS1, (b) DS2, (c) DS3, (d) DS4, (e) DS5, (f) DS6, (g) CD1, (h) CD2, (i) CD3.

Table S1 Performance of Different Classification Models on the Test Set.

Preprocessing	Wavelength selection algorithm	Models	Wavelength number	Accuracy (%)	Precision (%)	Recall (%)	F1 Score (%)
NP	–	PLS-DA	453	90.18	89.97	90.18	90.03
		SVM		91.99	92.21	91.99	92.05
		1D-CNN		89.66	91.97	89.66	90.81
		Googlenet		97.32	97.48	97.32	97.32
		EfficientNetV2		97.32	97.60	97.32	97.27
		DeiT		98.47	98.47	98.44	98.56
		DeiT-CBAM		99.62	99.63	99.62	99.62
	IRIV	PLS-DA	38	90.42	90.32	90.42	90.22
		SVM		91.99	92.64	91.95	92.11
		1D-CNN		91.57	92.66	91.57	91.64
		Googlenet		98.47	98.65	98.47	98.49
		EfficientNetV2		98.85	98.96	98.85	98.86
		DeiT		98.85	98.89	98.85	98.85
		DeiT-CBAM		99.23	99.23	99.23	99.23
	IVSO	PLS-DA	68	89.27	89.82	89.27	89.31
		SVM		90.42	90.60	90.42	90.42
		1D-CNN		91.57	93.98	91.57	92.74
		Googlenet		98.08	98.28	98.08	98.07
		EfficientNetV2		98.08	98.48	98.08	98.27
		DeiT		99.23	99.23	99.23	99.23
		DeiT-CBAM		98.85	98.86	98.85	98.85
	SPA	PLS-DA	79	90.42	90.27	90.42	90.08
		SVM		92.34	92.13	92.34	92.38
		1D-CNN		93.49	93.68	93.49	93.48
		Googlenet		98.47	98.47	98.47	98.44
		EfficientNetV2		95.79	96.34	95.79	95.74
		DeiT		98.85	98.86	98.85	98.85
		DeiT-CBAM		99.62	99.63	99.62	99.62
SG	–	PLS-DA	453	92.34	92.22	92.34	92.26
		SVM		94.25	94.71	94.25	94.27
		1D-CNN		90.80	92.18	90.80	91.47
		Googlenet		98.47	98.48	98.47	98.45
		EfficientNetV2		97.32	97.60	97.32	97.27
		DeiT		98.85	98.86	98.85	98.85
		DeiT-CBAM		99.23	99.23	99.23	99.23
	IRIV	PLS-DA	38	84.11	83.72	84.11	83.26
		1D-CNN		91.57	92.66	91.57	92.12
		SVM		87.08	87.33	87.08	87.15
		Googlenet		97.70	97.78	97.70	97.71
		EfficientNetV2		97.70	97.91	97.70	97.73
		DeiT		98.08	98.11	98.08	98.08
		DeiT-CBAM		98.85	98.86	98.85	98.85
	IVSO	PLS-DA	68	88.76	88.56	88.76	88.36
		SVM		92.72	93.08	92.72	92.70
		1D-CNN		92.34	92.78	92.34	92.56
		Googlenet		98.85	98.95	98.85	98.85
		EfficientNetV2		98.47	98.47	98.47	99.15
		DeiT		98.47	98.51	98.47	98.47
		DeiT-CBAM		99.23	99.28	99.23	99.23
	SPA	PLS-DA	94	90.04	89.89	90.04	89.82
		SVM		93.10	93.78	93.10	93.15
		1D-CNN		92.72	93.2	92.72	92.97
		Googlenet		98.47	98.48	98.47	98.45
		EfficientNetV2		93.10	94.57	93.10	92.94
		DeiT		98.08	98.08	98.08	98.06
		DeiT-CBAM		99.23	99.23	99.23	99.23

Note: "–" indicates no wavelength selection.

Table S2 Collection Sources of Danshen and Its Adulterants

Sample Codes	City	Province	Plant Name
DS1	Jinan	Shandong	Danshen (*Salvia miltiorrhiza*)
DS2	Nanyang	Henan	
DS3	Rizhao	Shandong	
DS4	Shangluo	Shaanxi	
DS5	Deyang	Sichuan	
DS6	Linyi	Shandong	
CD1	Panzhihua	Sichuan	*Dipsacus asperoides*
CD2	Zhaotong	Yunnan	*argentodoxa cuneata*
CD3	Zhaotong	Yunnan	*Arctium lappa*
